# Sirt1 Regulates Oxidative Stress in Oxygen-Glucose Deprived Hippocampal Neurons

**DOI:** 10.3389/fped.2020.00455

**Published:** 2020-08-14

**Authors:** Lina Shi, Jing Zhang, Yan Wang, Qingfei Hao, Haoming Chen, Xiuyong Cheng

**Affiliations:** ^1^Department of Neonatology, The First Affiliated Hospital of Zhengzhou University, Zhengzhou, China; ^2^Department of Pediatrics, Henan Medical College, Xinzheng, China

**Keywords:** hypoxic, ischemic, oxidative stress, pgc-1α, sirt1, resveratrol

## Abstract

Oxidative stress is an important mechanism of neonatal hypoxic-ischemic brain damage. Sirtuin1 (Sirt1) is a deacetylase that depends on NAD^+^, which has an important role in antioxidant metabolism. Furthermore, peroxisome proliferator-activated receptor γ-co-activator 1α (PGC-1α) is a key regulator of mitochondrial oxidative stress, which is regulated by Sirt1. Here, we investigated the role of Sirt1 in the pathogenesis of brain injuries after modulating its activity in primary cultured hippocampal neurons. Our study shows that the expression of Sirt1 was downregulated after oxygen-glucose deprivation. Activation of Sirt1 with resveratrol improved cell's resistance to oxidative stress, whereas inhibition of Sirt1 with EX527 significantly reduced cell viability after cellular oxidative stress. Our study also shows that activation of Sirt1 with resveratrol exerts its antioxidant effect by regulating the expression of PGC-1α. In contrast, application of EX527 decreased the expression of PGC-1α. In summary, these results confirmed that Sirt1 is a potent protective factor for neurons subjected to oxidative stress, and the protective effect of Sirt1 is attributed to its regulation of PGC-1α.

## Introduction

Although therapeutic hypothermia has been widely applied, 40–50% of Hypoxic ischemic encephalopathy (HIE) neonates still have some neurodevelopmental problems (1). Hypoxic ischemia (HI) involves multiple mechanisms, but the detailed pathogenesis is still unclear. Therefore, studies aiming to reducing morbidity and mortality of neonates with brain injuries are still needed.

Oxidative stress is one of the most important mechanisms of perinatal HI. After hypoxia and ischemia, the production of reactive oxygen species (ROS) rapidly accumulates and leads to mitochondrial dysfunction and delayed neuronal death. Oxidative stress-mediated mitochondrial damage has been shown to be involved in apoptosis.

Sirtuins are a class of NAD^+^-dependent histone deacetylases widely expressed in living organisms. Sirt1, also named as sirtuin1, is the most widely studied sirtuin has been an important therapeutic target in recent years. The translated protein has a molecular weight of about 60 kD and exhibits NAD^+^-dependent deacetylase activity. Previous studies showed that Sirt1 can regulate diabetes-induced cardiac dysfunction and brain ischemic reperfusion injuries by preventing mitochondrial dysfunction and alleviating hepatic steatosis (2–4). A large number *in vitro* and *in vivo* studies have shown that the activation of Sirt1 enhances mitochondrial biogenesis and augments oxidative metabolic capacity through different pathways (5). Studies also show that Sirt1 regulates apoptosis through other pathways, such as the anti-inflammatory pathway of NF-κB acetylation and the regulation of AMPK in autophagy (6, 7). Additionally, evidence has revealed that enhancing Sirt1 activity can reduce ROS production, reduce inflammation of neurons and glial cells, so as to reduce neuronal cell death (8). However, the role of Sirt1 in perinatal brain injuries is not completely understood.

Resveratrol (RSV) is a kind of polyphenol, a plant secondary metabolite extracted from wine, grain, fruit, root, etc., which has a protective effect on cardiovascular and nervous system diseases by regulating anti-inflammatory and anti-oxidation processes. Some studies have also reported that resveratrol is an activator of Sirt1 (9–11). According to a study on diabetic nephropathy by Tao Zhang et al., RSV can improve mitochondrial function, reduce oxidative stress in podocytes, and inhibit apoptosis induction through the Sirt1/PGC-1a axis (12). There is evidence in a brain injury model that RSV has a protective effect (13, 14). In contrast, EX527 is an effective selective Sirt1 inhibitor, which can inhibit Sirt1 deacetylase activity effectively. Maayan Waldman et al. showed that EX527 could increase ROS production by inhibiting Sirt1 activity, which aggravates myocardial injuries caused by diabetes (15). Another study on ischemic strokes also showed that EX527 can aggravate the expression of apoptotic proteins (16).

PGC-1α is a potent stimulator of mitochondrial biogenesis and respiration. It potentially increases neuroprotection while stimulating a broad anti-ROS response via mitochondrial function adaptation in neurological diseases such as Parkinson's disease, Alzheimer's disease, and brain trauma (17, 18). It has been shown that PGC-1α can be activated by deacetylation of Sirt1. Both play an important role in mitochondrial oxidative stress, including anti-oxidative stress, inhibition of inflammatory response, and reduction of apoptosis (19).

HI often causes damage to the hippocampus. In this study, based on a model of oxygen-glucose deprived primary cultured hippocampal neurons, we used RSV and EX527, an agonist and inhibitor of Sirt1, respectively, to investigate the mechanism and potential pathway of Sirt1 in HI.

## Materials and Methods

### Model Construction

#### Primary Culture of Hippocampal Neurons

All animal studies were approved by the Zhengzhou University committee for animal care and use for animal research. The experimental operation was performed according to the international guidelines for animal studies. Pregnant Sprague-Dawley rats were purchased from the Huaxing experimental animal farm (Zhengzhou, China). We followed an established protocol, obtaining rat embryonic hippocampal neurons from 18-day-old pregnant Sprague-Dawley rats. The hippocampus was detached under a microscope and then digested by trypsin (Hyclone, USA) at 37°C for 10–15 min. Single cells were obtained by blowing and centrifuging. The cell suspension was transferred into planting medium containing DMEM/high glucose (Hyclone, USA), 10% (v/v) fetal bovine serum (Hyclone, USA), 1% (v/v) glutamine (Hyclone, USA), and 1% (v/v) penicillin/streptomycin solution (Hyclone, USA), and subsequently plated on plates pretreated with poly-D-lysine (Sigma, USA). The density was 7 × 10^5^ cells/well in 6-well plates or 2 × 10^4^ cells/well in 96-well plates. After 4 h of incubation, the planting medium was replaced with an equal volume of maintenance medium containing neurobasal medium (Gibco, USA), 2% (v/v) B-27 (Gibco, USA), 1% (v/v) glutamine (Hyclone, USA), and 0.5% penicillin/streptomycin solution (Hyclone, USA). Half of the medium was replaced every 3 days.

#### Purity Identification

NeuN is a neuronal protein that is localized in the nuclei and the perinuclear cytoplasm of most neurons in the central nervous system of mammals (20). We used immunofluorescent NeuN antibodies that labeled the neurons in order to confirm their purity. The experiment was performed on the 7^th^ *in vitro* day. The coverslips were rinsed in PBS and the cells were fixed in 4% paraformaldehyde in PBS for 30 min. After being incubated in blocking solution (10% donkey serum and 0.06% Triton X-100 in PBS), the coverslips were treated with NeuN antibodies (Millipore, 1:100, USA) and FITC goat anti-mouse antibodies (Abclonal, 1:50, China) in diluting solution (0.01% Triton X-100 and 1% donkey serum in PBS). Then, the coverslips were mounted using DAPI (Vector Lab, Chicago) and the cells were observed under a microscope coupled to a camera. The ratio of the stained cytoplasm and nuclei represented the purity of the neuron.

#### The Oxygen-Glucose Deprivation Model

The oxygen glucose deprivation (OGD) model is a common cell level model to simulate neonatal hypoxic-ischemic brain damage. Although the time of hypoxia varies (21–23), our previous experiments showed that 30 min of hypoxia can build a successful model for hippocampal neurons (24). To induce OGD, we removed the maintaining medium and switched to DMEM without glucose (Gibco, USA). In this medium, cells were incubated at 37°C for 30 min in a sealed chamber, ventilated with 5% CO_2_/95% N_2_. At the end, the medium was replaced with normal medium. The cells were incubated for 12 h under normal conditions for subsequent experiments.

### RSV and EX527 Treatment and Effect

#### RSV and EX527 Treatment

Cells were divided into four groups during EX527 treatment (control, OGD, OGD+EX527, OGD+EX527+RSV) and three groups during RSV treatment (control, OGD, OGD+RSV). Cells were pretreated with 25 μM EX527 (MedChemExpress, USA) for 3 hours before OGD, and post-treated with 20 μM RSV (Solarbio, China) immediately after OGD.

#### MTT for Cell Viability

MTT reduces succinate dehydrogenase in mitochondria of living cells to water-insoluble blue-purple crystalline armor (Formazan) and deposits it in cells. As this process cannot happen in dead cells, it can be used to examine cell viability. Cells were plated into 96-well plates. We analyzed the cells 12 h after OGD induction. We added 10 μl MTT (Amresco, USA) to all groups and incubated the cells for 4 h. Then, the medium was removed and 100 μl DMSO (Amersco, USA) was added. Finally, we detected absorbance at 562 nm with a microplate reader. Cell viability was calculated as (100%) = (experiment group—blank)/(control group—blank) × 100%.

#### MDA and SOD Levels

After they were exposed to the different treatments, the neurons were harvested, sonicated, and centrifuged to collect the supernatant. The supernatant was used to examine the levels of SOD and Malondialdehyde (MDA). Cellular MDA content and total SOD activity were examined using commercial assay kits (Jiancheng Institute of Biotechnology, Nanjing, China) according to the manufacturer's instructions. The absorbance was read at 450 nm for SOD activity and at 532 nm for MDA content. The bicinchoninic acid disodium protein assay kit (Solarbio Biotechnology, China) was used to quantify the concentration of total protein.

#### Flow Cytometry for Cell Apoptosis

Phosphatidylserine, located inside the cell membrane, migrates to the outside of the cell membrane at the early stage of apoptosis. Phosphatidyl binding protein V (Annexin V) is a calcium-dependent phospholipid-binding protein with high affinity to phosphatidylserine. Therefore, the apoptotic cells can be detected by Annexin V and flow cytometry combined with PI rejection. The procedure was performed following the manufacturer's instruction (BD, USA). Cells were analyzed by flow cytometry within 1 h.

#### Gene Expression With Quantitative RT-PCR

Quantitative RT-PCR assays were performed to measure the mRNA levels of *Sirt1* and *PGC-1*α. Total RNA was extracted using TRIzol Reagent (Invitrogen, USA) according to the manufacturer's protocol. An aliquot of 1 μg of total RNA was reversely transcribed using PrimeScript RT Master Mix (Takara, Japan). Quantitative PCR was performed using TB Green Premix Ex Taq (Takara, Japan). The forward primer for *Sirt1* was ACTGGAGCTGGGGTTTCT and the reverse primer for *Sirt1* was CTTGAGGGTCTGGGGAGGT. The forward primer for *PGC-1*α was TTCCAACAAACACATGCAC and the reverse primer for *PGC-1*α was CGCATTTCTAAAGCACCAG. *GAPDH* was used as an internal reference to normalize the data. The forward primer for *GAPDH* was ACAGCAACAGGGTGGTGGAC and the reverse primer was TTTGAGGGTGCAGCGAACTT. The RT-PCR parameters were as follows: 95°C for 30 s, 40 cycles of 95°C for 30 s, 54°C for 30 s, and 72°C for 45 s. The CT values were recorded as primary data, normalized according to their corresponding internal references, and analyzed using the 2^−ΔΔCT^ method. The relative gene expression was noted as the final results and statistically analyzed.

#### Western Blot

Western blot analysis was performed to measure the protein levels of Sirt1, PGC-1α, and caspase-3 using a standard protocol. Equal amounts of total protein, along with 5 μl of molecular weight marker (Thermo Fisher, USA), were electrophoresed on SDS-PAGE gels. After being incubated with primary antibodies and secondary antibodies, we exploited the Chemiluminescent HRP Substrate (Millipore, USA) to visualize the target proteins (Sirt1, 1:2,000, Bioss, China; PGC-1α, Cell Signaling Technology, 1:1,000; caspase-3, 1:1,000, Cell Signaling Technology, USA; GAPDH and β-actin, 1:10,000, Abclonal, China). The molecular weights of Sirt1 and PGC-1α are 58 and 130 kD, respectively. Finally, we used an Amersham Imager 600 to take pictures and ImageJ to analyze the gray-scale values. Every protein was normalized to its corresponding internal reference. The ratios were noted as the final results and statistically analyzed.

### Statistics Analysis

All population data were expressed as means ± SD. Statistical analysis was performed by using GraphPad Prism (GraphPad Software). A *P* < 0.05 was considered significant.

## Results

### Successful Model Construction

#### Cell Purity

**Figure 1:**
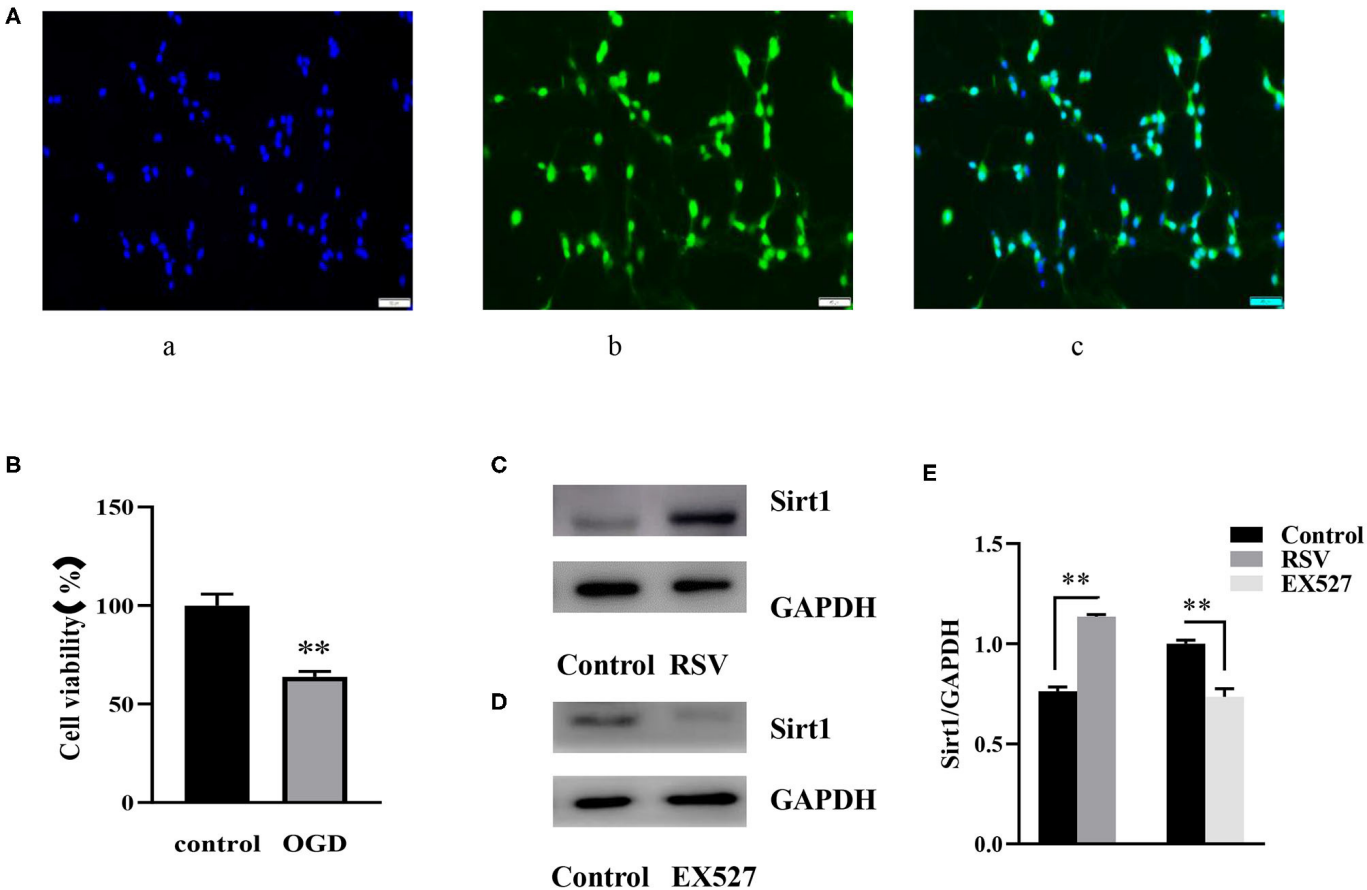
**(A)** Immunofluorescence of NeuN (400×). **(A)** (a) DAPI-stained nuclei (blue), **(A)** (b) NeuN staining identifies neurons (green), **(A)** (c) Merge image of a and b. A indicates the purity of the cultured cells. **(B)** Effects of OGD on cell viability were determined by an MTT assay (*n* = 6). **(C)** Western blot analysis of Sirt1 in the RSV (20 μM) group compared to the control group (*n* = 3). **(D)** Western blot analysis of Sirt1 in the EX527 (25 μM) group compared to the control group (*n* = 3). **(E)** Bar graph showing the relative *Sirt1* protein levels in each group. Data were obtained from three independent experiments. **P < 0.01.

As shown in Figure 1A (c), almost all blue cell nuclei (DAPI) are surrounded by green fluorescence (NeuN), indicating that almost all cultured cells were hippocampal neurons. The result showed that the cultured cells were of high purity and could be used in subsequent experiments.

#### Cell Viability Decreased After OGD

Twelve hours after OGD, cell viability of the control and OGD group was 100% and 63.785 ± 2.819%, respectively (Figure 1B). Compared to the control group, cell viability in the OGD group was significantly decreased, indicating that OGD was successfully induced.

#### Sirt1 Was Downregulated After OGD

**Figure 2:**
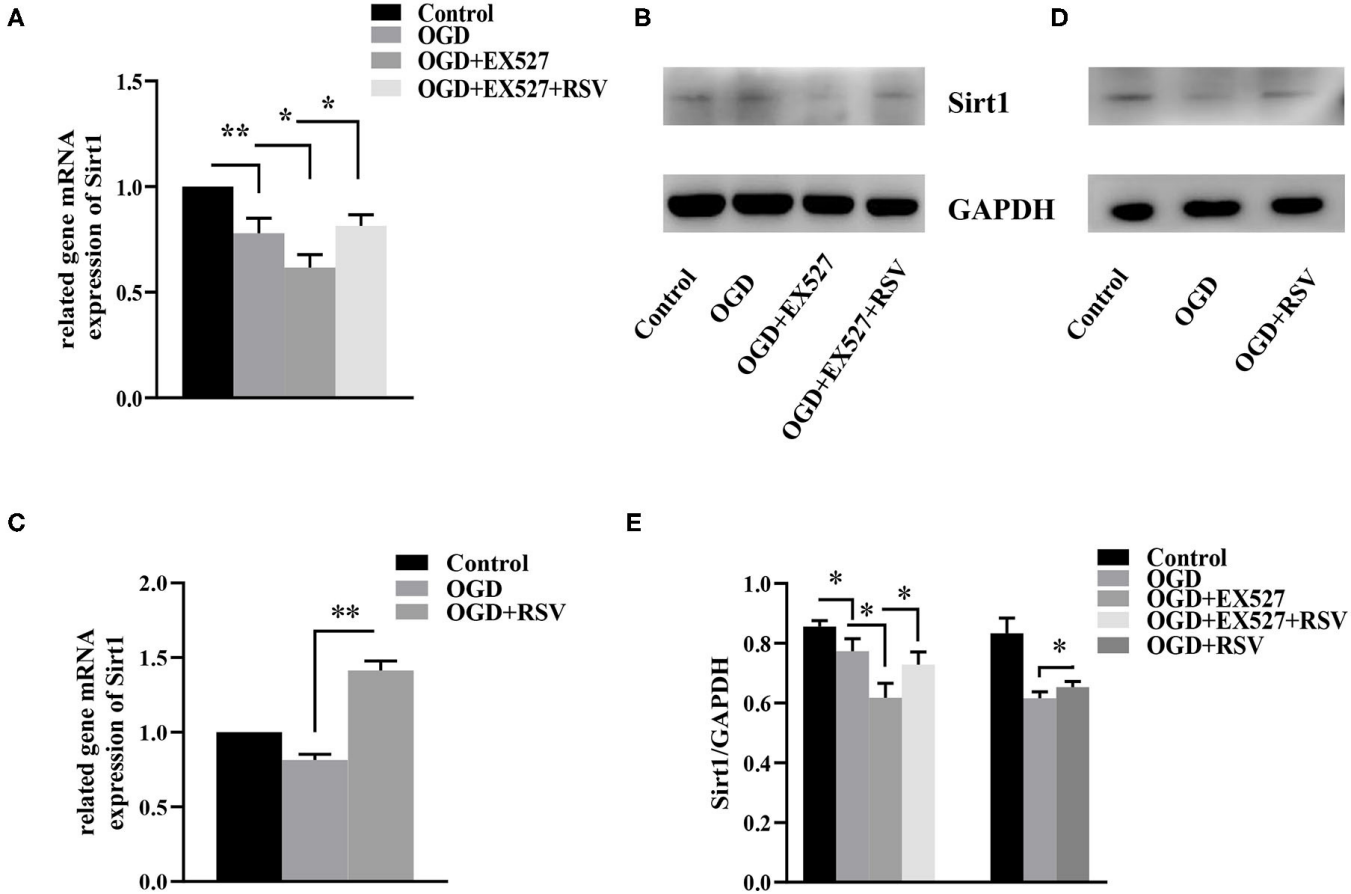
**(A)** Relative *Sirt1* mRNA levels in different groups after EX527 treatment (*n* = 3). **(B)** Relative Sirt1 protein levels in different groups after EX527 treatment (*n* = 3). **(C)** Relative *Sirt1* mRNA levels in different groups after RSV treatment (*n* = 3). **(D)** Western blot analysis of Sirt1 in different groups after RSV treatment (*n* = 3). **(E)** Bar graph showing the relative *Sirt1* protein levels in each group. Data were obtained from three independent experiments. *P < 0.05, **P < 0.01.

The relative *Sirt1* mRNA levels in the control and the OGD group were 1 and 0.780 ± 0.070, respectively. Compared to the control group, the relative expression in the OGD group was decreased (*P* < 0.01). Sirt1 protein levels were also lower in the OGD group than in the control group (0.856 ± 0.020 vs. 0.773 ± 0.042, *P* < 0.05, Figures 2A,B).

### Effects of EX527 and RSV at Specific Concentrations on Sirt1 Expression in Normal Cells

After preliminary experiments, we chose to apply 25 μM EX527 3 h before the OGD induction, and 20 μM RSV immediately after OGD. At this concentration, RSV upregulated the expression of Sirt1 protein without affecting cell viability (0.763 ± 0.022 vs. 1.135 ± 0.010, *P* < 0.01, Figure 1C). EX527 downregulated the expression of Sirt1 (1.000 ± 0.017 vs. 0.735 ± 0.040, *P* < 0.01, Figure 1D). The relative Sirt1 protein levels in each group was showed in Bar graph (Figure 1E). Therefore, they can be used to increase or decrease Sirt1 activity, respectively, for subsequent experiments.

### EX527 Treatment Effects After OGD

#### Effect of EX527 on Sirt1 Expression After OGD

The relative levels of *Sirt1* mRNA in the control group, the OGD group, the OGD+EX527 group, and the OGD+EX527+RSV group were 1, 0.780 ± 0.070, 0.620 ± 0.062, and 0.815 ± 0.052, respectively (Figure 2A). Sirt1 protein levels were 0.856 ± 0.020, 0.773 ± 0.042, 0.618 ± 0.047, and 0.728 ± 0.042, respectively (Figure 2B). The relative Sirt1 protein levels in different groups after EX527 treatment was showed in Bar graph (Figure 2E). Compared to the OGD group, the relative levels of both Sirt1 mRNA and protein in the OGD+EX527 group were decreased (*P* < 0.05, *P* < 0.05). The mRNA and protein levels in the OGD+EX527+RSV group were higher than in the OGD+EX527 group (*P* < 0.05, *P* < 0.05). So, EX527 can reduce Sirt1 expression after OGD.

#### Pretreatment of EX527 Increased Apoptosis and Decreased Cell Viability After OGD

**Figure 3:**
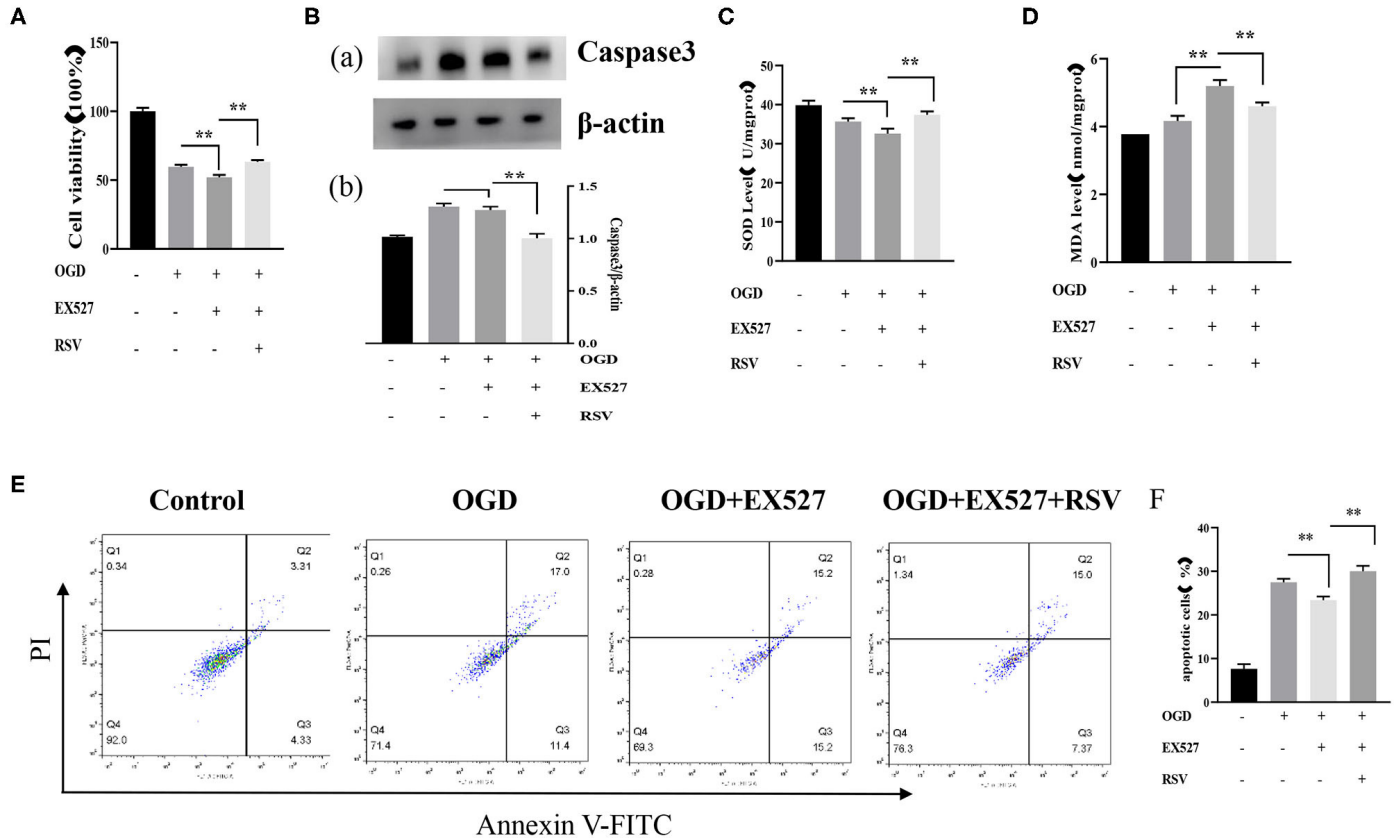
**(A)** Effects of EX527 treatment on the viability of OGD-treated cells was determined by the MTT assay (*n* = 6). **(B)** (a) Western blot analysis of Caspase-3 in different groups (*n* = 3). (b) Bar graph showing the relative *Caspase-3* protein levels in each group. **(C)** Intracellular SOD levels in different groups (*n* = 6). **(D)** Intracellular MDA levels in different groups (*n* = 6). **(E)** Flow cytometry analysis of apoptotic neurons with different treatments. **(F)** Flow cytometry analysis of apoptotic cells with different treatments (*n* = 6). Data were obtained from three independent experiments **P < 0.01.

Apoptosis was detected via MTT, caspase-3, and Annexin V-FITC/PI. The cell viability in the OGD+EX527 group was lower than in the OGD group as detected via the MTT method (59.694 ± 1.524% vs. 52.062 ± 1.805%, *P* < 0.01, Figure 3A). Figure 3E showed the apoptosis in different groups after EX527 treatment. The apoptosis rate of the OGD+EX527 group was higher than in the OGD group as detected by flow cytometry analysis (27.267 ± 0.804% vs. 30.000 ± 1.251%, *P* < 0.01, Figure 3F). Oxidative stress results showed that EX527 weakened the protection of ROS. The intracellular SOD level of the OGD+EX527 group was lower than in the OGD group (35.692 ± 0.833 U/mgprot vs. 32.598 ± 1.272 U/mgprot, *P* < 0.01, Figure 3C). The intracellular MDA content of the OGD+EX527 group was higher than in the OGD group (4.166 ± 0.149 nmol/mgprot vs. 5.196 ± 0.176 nmol/mgprot *P* < 0.01, Figure 3D). However, the expression of caspase-3 in the OGD+EX527 group did not increase significantly compared to the OGD group (1.304 ± 0.300 vs. 1.272 ± 0.033, *P* > 0.05, Figure 3B). Conversely, RSV increased Sirt1 expression and cell viability. As shown in Figure 3, compared to the OGD + EX527 group, the cell viability and oxidative stress indices of the OGD + EX527 + RSV group had corresponding changes.

### RSV Treatment Effects After OGD

#### Effect of RSV on Sirt1 Expression After OGD

The relative levels of *Sirt1* mRNA in the control group, the OGD group, and the OGD+RSV group were 1, 0.814 ± 0.038, and 1.415 ± 0.064, respectively (Figure 2C). The protein levels were 0.833 ± 0.051, 0.609 ± 0.011, and 0.653 ± 0.019, respectively (Figure 2D). The relative Sirt1 protein levels in different groups after RSV treatment was showed in Bar graph (Figure 2E). Compared to the OGD group, Sirt1 mRNA and protein levels were increased in the OGD+RSV group, indicating that RSV can increase Sirt1 expression after OGD (*P* < 0.01, *P* < 0.05).

#### RSV Treatment Decreased Apoptosis and Increased Cell Viability After OGD

**Figure 4:**
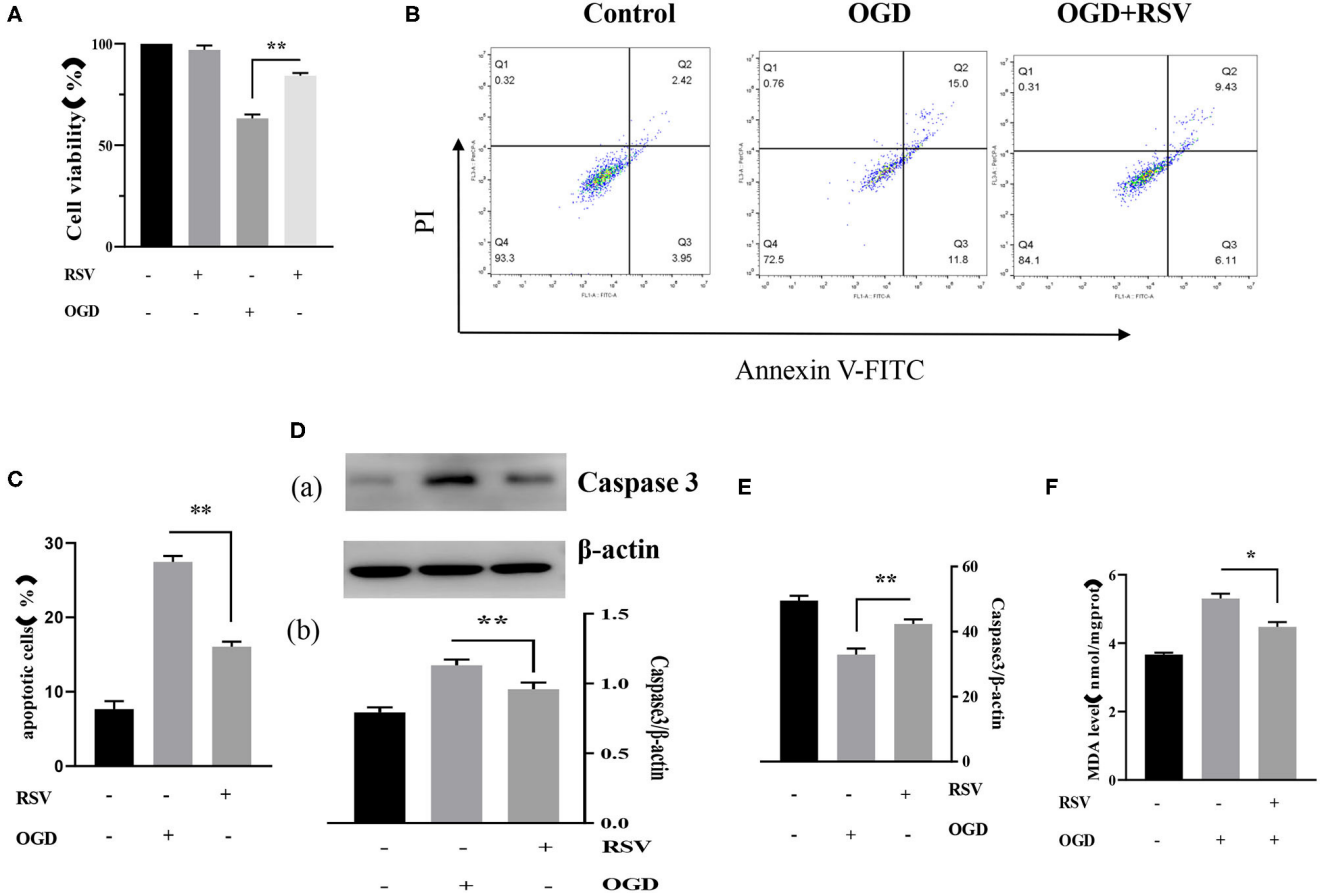
**(A)** Effects of RSV treatment on the viability of OGD-treated cells was determined by the MTT assay (*n* = 6). **(B)** Flow cytometry analysis of apoptotic neurons with different treatments. **(C)** Flow cytometry analysis of apoptotic cells with different treatments (*n* = 6). **(D)** (a) Western blot analysis of Caspase-3 in different groups (*n* = 3). (b) Bar graph showing the relative *Caspase-3* protein levels in each group. **(E)** Intracellular SOD levels of different groups (*n* = 6). **(F)** Intracellular MDA levels of different groups (*n* = 6). Data were obtained from three independent experiments. *P < 0.05, **P < 0.01.

Apoptosis was examined in the same way as described above. Our experiments showed that RSV treatment decreased apoptosis. The cell viability of the OGD+RSV group was higher than in the OGD group, as detected by the MTT method (63.238 ± 1.931% vs. 84.240 ± 1.322%, *P* < 0.01, Figure 4A). Figure 4B showed the apoptosis in different groups after RSV treatment. The apoptosis rate of the OGD+RSV group was lower than in the OGD group, detected by flow cytometry analysis (27.267 ± 0.804% vs. 16.951 ± 0.679%, *P* < 0.01, Figure 4C). The results showed that RSV treatment could reduce caspase-3-mediated apoptosis (1.130 ± 0.041 vs. 0.960 ± 0.470, *P* < 0.01, Figure 4D). Intracellular SOD and MDA levels are common indicators of oxidative stress. Our experiments showed that RSV could reduce intracellular oxidative stress. The intracellular SOD level of the OGD+RSV group was higher than in the OGD group (32.904 ± 1.891 U/mgprot vs. 42.339 ± 1.393 U/mgprot, *P* < 0.01, Figure 4E). The intracellular MDA levels of the OGD+RSV group were lower than of the OGD group (5.307 ± 0.141 nmol/mgpro vs. 4.477 ± 0.139 nmol/mgprot, *P* < 0.01, Figure 4F).

#### RSV Pretreatment Was Also Protective for OGD

**Figure 5:**
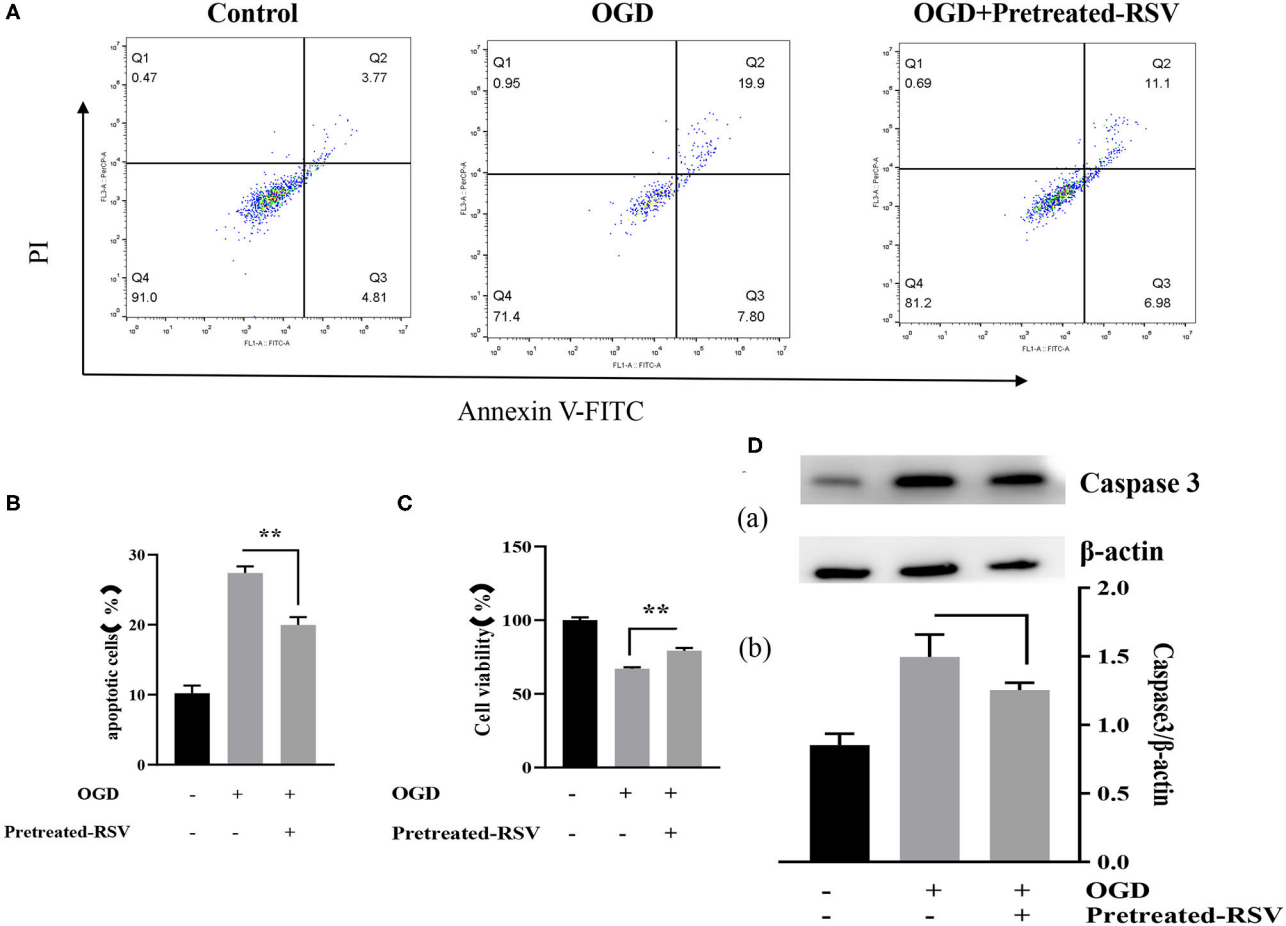
**(A)** Flow cytometry analysis of apoptotic cells with different treatments. **(B)** Apoptotic cells in different groups are shown as mean ± SD (*n* = 6). **(C)** Cell viability of pretreated-RSV cells compared to the OGD group (*n* = 6). **(D)** (a) Western blot analysis of Caspase-3 in different groups (n = 3). (b) Bar graph showing the relative *Caspase-3* protein levels in each group. Data were obtained from three independent experiments. **P < 0.01.

To further investigate the expression of Sirt1, we analyzed protective effects by adding RSV 4 h before OGD. We performed flow cytometry analysis and apoptosis-related protein expression analysis to test the potential benefit of RSV application. Figure 5A showed the apoptosis in different groups after RSV pretreatment. The apoptosis rate of the OGD+pretreated RSV group was lower than of the OGD group as assessed by flow cytometry analysis (27.397 ± 0.942% vs. 19.952 ± 1.130%, *P* < 0.01, Figure 5B). The cell viability of the OGD+pretreated RSV group was higher than in the OGD group as detected with the MTT method (67.012 ± 1.055% vs. 79.277 ± 1.892%, *P* < 0.01, Figure 5C). Therefore, increasing Sirt1 activity before OGD can also reduce apoptosis. However, we did not detect a decrease in caspase-3 expression (1.497 ± 0.163 vs. 1.253 ± 0.054, *P* > 0.05, Figure 5D).

### Sirt1 Was Involved in Apoptosis by Regulating PGC-1α Levels

**Figure 6:**
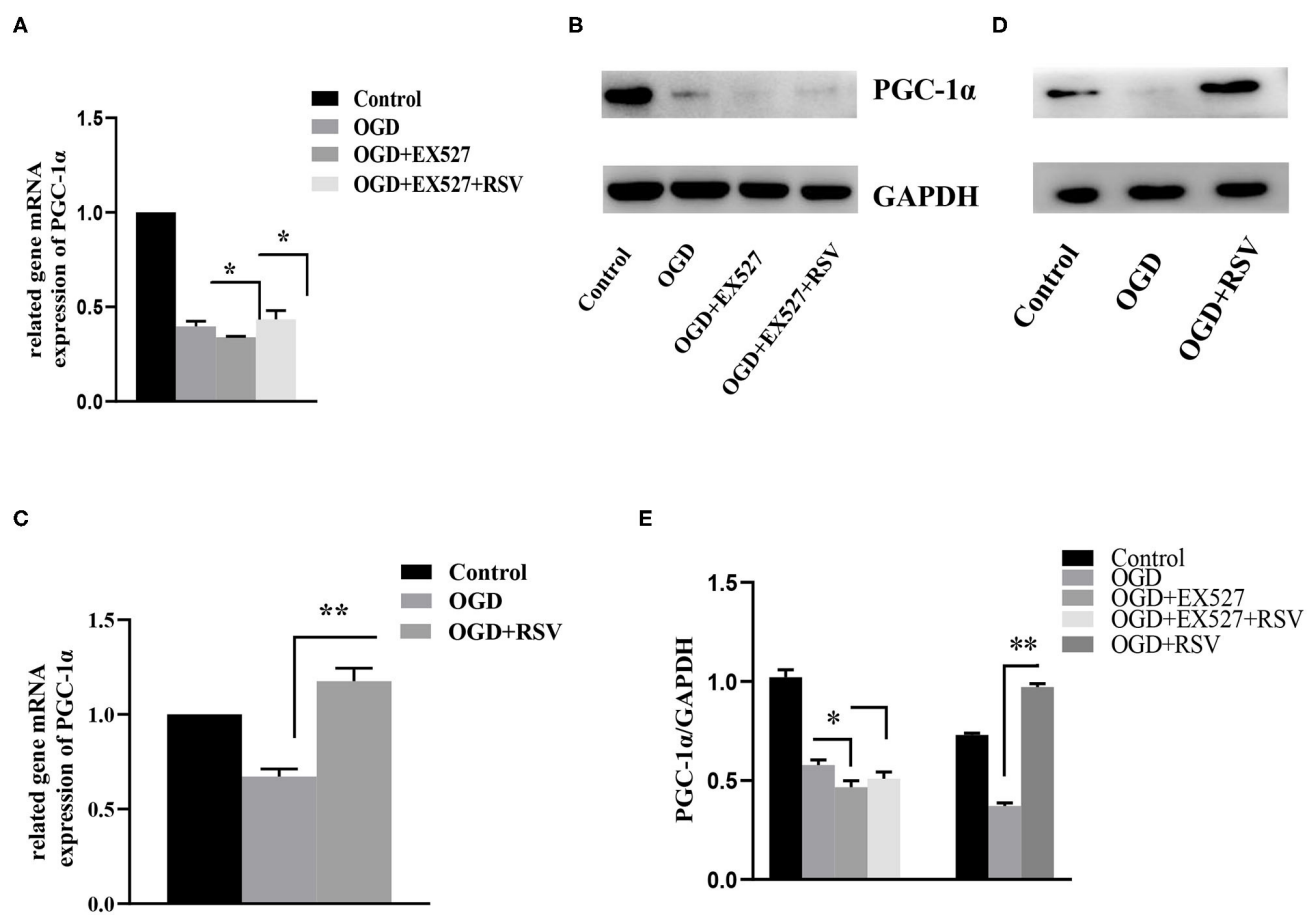
**(A)** Relative *PGC-1*α mRNA levels in different groups after EX527 treatment (*n* = 3). **(B)** Relative PGC-1α protein levels in different groups after EX527 treatment (*n* = 3). **(C)** Relative *PGC-1*α mRNA levels in different groups after RSV treatment (*n* = 3). **(D)** Relative PGC-1α protein levels in different groups after RSV treatment (*n* = 3). Data were obtained from three independent experiments. **(E)** Bar graph showing the relative expression of PGC-1α in each group. *P < 0.05, **P < 0.01.

PGC-1α is regulated by Sirt1 and is involved in mitochondrial oxidative stress. Therefore, we also tested the change in the expression of PGC-1α after addition of RSV or EX527. As shown in Figure 6, compared to the OGD group, the relative levels of Sirt1 and PGC-1α mRNA and protein levels in the OGD+EX527 group were decreased. Compared to the OGD+EX527 group, the relative mRNA levels of *Sirt1* and *PGC-1*α in the OGD+EX527+RSV group were both increased. However, although the relative expression levels of Sirt1 protein was increased, PGC-1α protein levels were not significantly increased, which was mainly because the changes of PGC-1α protein were not synchronized at the time point we selected.

## Discussion

Despite the fact that therapeutic hypothermia is an effective treatment in hypoxic ischemia, neonatal brain injury is still one of the main reasons for morbidity and mortality in children (25). Studies in previous decades have shown that reperfusion injury and delayed programmed cell death (apoptosis) of neurons are important pathophysiological mechanisms (26–28). ROS destroy proteins, nucleic acids, and membrane polyunsaturated fatty acids, lead to lipid peroxidation and loss of membrane integrity, reduce mitochondrial membrane potential, increase the membrane permeability to Ca^2+^, and eventually lead to neuron apoptosis (29–31).

Sirt1 is a member of the sirtuin family, which is a crucial epigenetic regulator involved in many biological and pathological processes including metabolism, genomic stability maintenance, immune responses, and others (32, 33). Earlier studies have shown that Sirt1-related signal pathways mediate oxidative stress in brain injury (34–37). Sirt1 is also an upstream protein of PGC-1α. Therefore, we mainly analyzed the effect of the Sirt1/PGC-1α signal pathway on oxidative stress and neurocytes to investigate its neuroprotective effects in hypoxia/reperfusion.

In our work, we found that Sirt1 expression decreased after OGD, while apoptosis increased and cell viability decreased, indicating that Sirt1 may be related to neuronal apoptosis after OGD. Surprisingly, after adding EX527, a Sirt1 inhibitor, we found that neuronal apoptosis was increased and cell viability was decreased, confirming that Sirt1 was involved in cell apoptosis. Next, when adding RSV, a Sirt1 agonist, we found that neuron apoptosis was decreased and cell viability was increased. Therefore, we hypothesize that Sirt1 may be neuroprotective, which is consistent with results from a previous *in vivo* study (38). Moreover, RSV showed a neuroprotective effect either before or after OGD, indicating its great potential for the treatment of brain injury.

Mitochondria play a key role in regulating cellular calcium levels and activating cell death pathways. The Sirt1/PGC-1α pathway plays an important role in mitochondrial function regulation. Sirt1 can directly regulate PGC-1α activity through phosphorylation and deacetylation. And PGC-1α further induce mitochondrial gene expression in neurons to coordinate energy metabolism, enhance activity, and further regulate mitochondrial biogenesis and function, so as to reduce oxidative stress-mediated neuronal death. The Sirt1/PGC-1 pathway can play a protective role by activating autophagy against oxidative stress-mediated ROS production in systemic endogenous stress syndrome (39). In diabetic peripheral neuropathy, overexpression of Sirt1 can increase axonal growth through Sirt1/ PGC-1α/ TFAM axis and improve mitochondrial oxidative metabolism (40). It has also been shown that Sirt1/PGC-1α signaling pathway can further regulate the role of uncoupling protein 2(UCP2) or Forkhead box protein O1(FOXO1) in reducing oxidative stress and neuronal apoptosis (41, 42). In our study, decreasing Sirt1 reduced the expression of PGC-1α and increased apoptosis, resulting in intracellular increased MDA and decreased SOD, which are related to cell apoptosis. Therefore, we hypothesize that the neuroprotective effect of Sirt1 in neonatal brain injury is partly exerted by activating PGC-1α.

Although our experiments showed that Sirt1 is involved in the regulation of OGD, the described experiments were only performed *in vitro*. We do not know if the regulatory role described above is the same as *in vivo*. In addition, we selected a single time point after OGD. The trend in Sirt1 changes at different time points is out of scope of this study. Furthermore, the long-term protective effects of Sirt1 after OGD remain unclear, too.

In conclusion, oxidative stress is important for the occurrence of hypoxic brain injury. The antioxidant effect of Sirt1 may be a target of future HI studies. Our study confirms that Sirt1 is involved in apoptosis and is a protective antioxidant, which is regulated by the Sirt1/PGC-1α signaling pathway. Therefore, Sirt1 may serve as an important target in future studies for effective treatments of neonatal brain injury.

## Data Availability Statement

The raw data supporting the conclusions of this article will be made available by the authors, without undue reservation.

## Ethics Statement

The animal study was reviewed and approved by Zhengzhou University committee on animal care and use for animal research.

## Author Contributions

XC designed the project. LS and JZ carried out experiments, analyzed the data, wrote the first draft, and made the final draft. YW and QH participated in the design of the project and revised the manuscript. HC helped perform the experiment. All authors reviewed the manuscript.

## Conflict of Interest

The authors declare that the research was conducted in the absence of any commercial or financial relationships that could be construed as a potential conflict of interest.
